# Isolation, Identification, and Pathogenicity of *Vibrio gigantis* Retrieved from European Seabass (*Dicentrarchus labrax*) Farmed in Türkiye

**DOI:** 10.3390/ani13223580

**Published:** 2023-11-20

**Authors:** Sevdan Yilmaz, Süheyla Karataş, Terje Marken Steinum, Mert Gürkan, Dilek Kahraman Yilmaz, Hany M. R. Abdel-Latif

**Affiliations:** 1 Department of Aquaculture, Faculty of Marine Sciences and Technology, Çanakkale Onsekiz Mart University, Çanakkale 17100, Turkey; 2 Department of Aquaculture and Fish Diseases, Faculty of Aquatic Sciences, Istanbul University, Istanbul 34134, Turkey; skaratas@istanbul.edu.tr; 3 Department of Molecular Biology and Genetics, Faculty of Sciences, Istanbul University, Istanbul 34134, Turkey; terje.steinum@istanbul.edu.tr; 4 Department of Biology, Faculty of Arts and Sciences, Çanakkale Onsekiz Mart University, Çanakkale 17100, Turkey; mertgurkan@comu.edu.tr; 5 Department of Marine Biology, Faculty of Marine Sciences and Technology, Çanakkale Onsekiz Mart University, Çanakkale 17100, Turkey; dilek.kahramanyilmaz@comu.edu.tr; 6 Department of Poultry and Fish Diseases, Faculty of Veterinary Medicine, Alexandria University, Alexandria 22758, Egypt

**Keywords:** *Dicentrarchus labrax*, histopathology, mortalities, vibriosis, *Vibrio gigantis*

## Abstract

**Simple Summary:**

*Vibrio gigantis* was first identified as an opportunistic pathogen of European seabass broodstock in Türkiye. The bacterium was isolated from the reproductive organs, liver, and spleen of diseased fish. A representative isolate C24 was unambiguously identified as *V. gigantis* based on high whole-genome average nucleotide identity values (ANI > 97.7%). Despite causing notable problems in broodstock, the *V. gigantis* C24 isolate exhibited low to moderate virulence in experimentally challenged juvenile European seabass.

**Abstract:**

In this study, *V. gigantis* strain C24 was isolated from cases of winter mortalities of hatchery-reared European seabass (*Dicentrarchus labrax*) broodstock in Türkiye. The first mortalities were reported in September 2016 and occurred annually in early autumn/late winter until the end of February 2019, when 15% of accumulated mortality was recorded. Diseased moribund fish exhibited general septicemic signs, including dermal ulcerations with hemorrhagic margins, distended abdomens, and hemorrhages below the pectorals, pelvic fins, and at the operculum. Postmortem findings showed congestion in several internal organs, hemorrhagic ascitic fluid, and congested prolapsed anal openings. The representative bacterial isolate *V. gigantis* strain C24 was characterized as Gram-negative, motile, nitrite-producing, and as vibrio static agent O/129-sensitive. The full-length 16S rRNA sequence (Accession No. ON778781) and *gyrB* gene sequence (Accession No. ON792326) of the C24 strain showed high similarity to *V. gigantis* strains. Moreover, the whole-genome average nucleotide identity (ANI) values (ANI > 97.7%) against four *V. gigantis* strains above the species demarcation limit unambiguously identified the C24 isolate as a member of this species. A preliminary virulence-gene analysis showed that the *V. gigantis* isolate C24 encoded at least three exotoxins, including two aerolysins and a thermolabile hemolysin. The experimental infection showed that the C24 isolate exhibited low to moderate virulence in experimentally infected European seabass juveniles. Interestingly, antimicrobial susceptibility testing revealed that the C24 isolate was susceptible to nalidixic acid, ciprofloxacin, and several other antibiotics but resistant to tilmicosin, kanamycin, streptomycin, and ampicillin. To our knowledge, this study is the first to report that *V. gigantis* could be considered an emerging bacterial pathogen in Türkiye, and it may threaten the international European seabass production.

## 1. Introduction

Aquaculture is one of the fastest-growing food production sectors, contributing more to global food production than capture fisheries. In recent years, aquaculture activities in Türkiye have greatly expanded, especially with the intensive marine aquaculture sectors because of the Mediterranean coasts [1]. There are several cultured marine fish species in Türkiye, including the gilthead seabream (*Sparus aurata*) and European seabass (*Dicentrarchus labrax*), and the Turkish production volume of these two species has been steadily increasing, rising from approx. 33.000 tons to 258.500 tons between 2000 and 2020 [2].

Bacterial fish diseases contribute to severe economic losses worldwide [3,4,5]. Vibriosis is a bacterial fish disease affecting a wide range of economically important farmed shrimp, marine fish species, and some freshwater fish around the globe [6,7,8]. Moreover, it has been recognized that vibriosis is responsible for economic losses and mass mortality events in several aquaculture species [9]. The genus *Vibrio* comprises several ubiquitous bacteria dispersed in aquatic environments and includes about 175 species, according to the List of Prokaryotic names withstanding in Nomenclature [10]. However, only some of them, primarily *Vibrio anguillarum*, *V. vulnificus*, *V. harveyi*, *V. ordalii*, *V. parahaemolyticus*, and *V. alginolyticus*, cause disease and mortalities in cultured fish species, including in gilthead seabream and European seabass [11,12]. A recent report revealed that *V. anguillarum* and *V. alginolyticus* are the predominant *vibrio* species widely dispersed in Turkish fish farms [13]. Several newly identified bacterial species of this genus are associated with marine organisms like mollusks, such as *V. crassostreae*, *V. gigantis*, *V. breoganii*, and *V. celticus* [14].

Bacterial fish diseases contribute to severe economic losses worldwide [3,4,5]. Vibriosis is a bacterial fish disease affecting a wide range of economically important farmed shrimp, marine fish species, and some freshwater fish around the globe [6,7,8]. Moreover, it has been recognized that vibriosis is responsible for economic losses and mass mortality events in several aquaculture species [9]. The genus *Vibrio* comprises several ubiquitous bacteria dispersed in aquatic environments and includes about 175 species, according to the List of Prokaryotic names withstanding in Nomenclature [10]. However, only some of them, primarily *Vibrio anguillarum*, *V. vulnificus*, *V. harveyi*, *V. ordalii*, *V. parahaemolyticus*, and *V. alginolyticus*, cause disease and mortalities in cultured fish species, including in gilthead seabream and European seabass [11,12]. A recent report revealed that *V. anguillarum* and *V. alginolyticus* are the predominant *vibrio* species widely dispersed in Turkish fish farms [13]. Several newly identified bacterial species of this genus are associated with marine organisms like mollusks, such as *V. crassostreae*, *V. gigantis*, *V. breoganii*, and *V. celticus* [14].

*V. gigantis* belongs to the Splendidus clade, which is the largest clade in the family *Vibrionaceae* [15]. This bacterium was first described in aquatic invertebrates. In 2005, *V. gigantis* was isolated from the hemolymph of cultured oysters (*Crassostrea gigas*) and characterized and identified using polyphasic analysis [16]. This bacterium was also isolated earlier from aquatic invertebrates such as sea cucumber species (*Apostichopus japonicus*) from the Great Bay near the Sea of Japan [17]. Moreover, *V. gigantis* was involved in skin ulceration disease in another sea cucumber species (*Holothuria arguinensis*) in southern Portugal [18]. Until now, only three reports concerning *V. gigantis* infection in finfish species have been published, as this bacterium was isolated from diseased olive flounder (*Paralichthys olivaceus*) farmed in South Korea [19], diseased dark-banded rockfish (*Sebastes inermis*) farmed in Korea [20], and recently reported on in relation to gilthead seabream (*Sparus aurata*) in Greece [21]. However, this bacterium was regarded as non-pathogenic or as low virulent to aquatic animals, and its pathogenic mechanisms and virulence factors have not yet been elucidated, warranting further investigations.

The main purpose of the present study was to investigate the causative agent of re-occurring disease outbreaks with mortalities in European seabass broodstock at a Turkish hatchery in the Çanakkale province, Türkiye. The initial observations during the last outbreak led to a preliminary diagnosis of a natural case of vibriosis. To this end, herein, we describe the clinicopathological findings, histopathological lesions, phenotypic characterization, and molecular identification of the etiological agent for accurately identifying and characterizing the *Vibrio* species involved in this outbreak. A strain of *V. gigantis* isolate C24 was identified. Some few putative virulence factors of this bacterial isolate were determined. Moreover, the antibiogram (antibiotic resistance) of this isolate has also been assessed. An experimental challenge was also carried out to validate the virulence of the *V. gigantis* isolate C24 in European seabass juveniles.

## 2. Materials and Methods

### 2.1. Case History, Outbreak, and Farm Conditions

The first onset of fish deaths occurred in September 2016 at a European seabass hatchery (40°16′31.6″ N 26°36′08.1″ E) within the borders of the Çanakkale province, Türkiye. The brood fish (20 kg/m^3^) were cultured in re-circulated aerated seawater. They were bred at a sex ratio of three females to two males (3♀:2♂). A total of 1.4 tons of seawater was changed daily at a rate of ∼10% of the total volume of tanks. The mortality of female and male brood fish in the facility peaked between November and December 2016. Disease outbreaks re-occurred yearly at the same time until the end of the final incidence in February 2019. It was noticed that fish deaths increased particularly when the seawater temperature dropped below 18 °C during the winter season. During this epidemic, the measured water quality parameters varied as follows: water temperature range—14–19 °C, dissolved oxygen—7.0–9.0 mg/L, pH values—7.8–8.2, and salinity—38–40 ppm.

### 2.2. Fish Sampling

Samples of European seabass broodstock showing gross clinical signs were collected using a handheld net. Moribund fish of both sexes (4–7 kg body weight and 3–5 years old) were sampled throughout the disease outbreak from September 2016 to February 2019. A total of 95 fish were sampled and investigated during the outbreak. A sum of 15% accumulated mortality was recorded in brood fish. These fish were first anesthetized (50 mg/L clove oil; Sigma Aldrich, St. Louis, MO, USA) and then transported alive in tanks supplied with oxygen to the laboratory in the Çanakkale province. Less than 4 h elapsed between sampling and the start of the laboratory investigations. The fish were killed with an overdose of anesthetic (250 mg/L clove oil) and immediately subjected to complete clinicopathological and bacteriological examinations. All procedures involving live fish followed the standard good practices for animal and fish welfare.

### 2.3. Clinical Examination (Gross Observations and Necropsy Findings)

Gross observations were performed on the collected moribund fish at the time of arrival at the laboratory. Any external signs of abnormality observed on the body surfaces of fish (*n* = 95) were recorded. At the same time, a necropsy was performed for evaluation of the postmortem (PM) lesions according to the procedures provided in previous publications [22,23].

### 2.4. Histopathological Examination

Liver, spleen, and intestine tissues were dissected from the examined broodstock fish and fixed in Bouin’s solution. Afterwards, the tissues were dehydrated in a progressive series of ethanol and embedded in paraffin. The tissues were cut to a 5 µm thickness using a Leica rotary microtome. The sections were stained with hematoxylin-eosin [24]. Histopathological changes were evaluated, and images were taken using a CX31 Olympus light microscope equipped with a digital camera using DP2-BSW (ver.2.1) software.

### 2.5. Bacterial Isolation Protocol

For bacterial isolation, samples from the reproductive organs, liver, and spleen of each fish were streaked directly onto Tryptic Soya Agar (TSA; Merck, Darmstadt, Germany) plates with 1.5% NaCl, Marine Agar (MA) (Difco™, Le Pont-de-Claix, France), and Thiosulfate Citrate Bile Sucrose Agar (TCBS; Merck, Darmstadt, Germany) plates and incubated at 22 °C for 48 h. A representative colony was picked from each agar plate and re-streaked onto new TSA+1.5% NaCl, TCBS, and MA plates to ensure a pure culture. Single colony and bacterial cell morphology were determined with the aid of Gram staining. All pure bacterial isolates (*n* = 305) were stored at −80 °C in tryptic soya broth tubes supplemented with 1.5% NaCl and 20% (*v*/*v*) glycerol for subsequent phenotypical characterization.

### 2.6. Phenotypic Characterization

The phenotypic characterization of the retrieved bacterial isolates was performed using a series of biochemical tests. These tests were carried out using commercially available API 20E kits (BioMérieux, Craponne, France). The carbohydrate metabolism profile of the bacterial isolate was carried out using API^®^ 50 CH kits (BioMérieux, France). These tests were performed according to the manufacturer’s protocol with one modification—2% NaCl was added to the freshly prepared bacterial suspension. After inoculation, the strips were incubated for 48 h at 20 °C, and the results were determined according to the manufacturer’s instructions. The bacterial sensitivity to vibrio static agent O/129 (150 μg) was determined using Oxoid discs (Basingstoke, UK).

### 2.7. Molecular Identification/Characterization of Bacterial Isolates

Pure cultures of 10 bacterial isolates were sent to BM Laboratuvar Sistemleri (Ankara, Türkiye) for genomic DNA extraction using the GeneMATRIX bacterial and yeast genomic DNA purification kit (EURx Ltd., Gdansk, Poland). The genomic DNA from these isolates was used as a template for 16S rRNA gene amplification using primer set 27F 5′-AGAGTTTGATCMTGGCTCAG-3′ and 1492R 5′-TACGGYTACCTTGTTACGACTT-3′ [25] in a standard PCR with FIREPol^®^ DNA Polymerase (Solis Biodyne, Tartu, Estonia) according to the manufacturer’s recommendations. The resultant PCR products were cleaned using the “HighPrep™ PCR Clean-up System” (MagBio Genomics Inc., Gaithersburg, MD, USA) before Sanger sequencing was carried out on the ABI 3730XL platform (Applied Biosystems, Foster City, CA, USA). A representative isolate (C24) was also full-genome sequenced on the Illumina Novaseq 6000 platform. The assembly of the draft genome was carried out using the SPAdes assembler [26], and the initial annotation of the draft genome using PROKKA [27] was performed by BM Laboratuvar Sistemleri (Ankara, Türkiye). For the preliminary identification of vibrio species, full-length housekeeping gene (16S rRNA and *gyrB*) sequences have been deposited in the NCBI GenBank database under Accession No. ON778781 and Accession No. ON792326, respectively.

The FastANI v1.33 software [28] was used to calculate the ANI values between the unfinished draft genome of the C24 isolate and a dataset comprising 191 genomes representing 122 bacterial species in the genus *Vibrio*. This dataset was constructed using data downloaded from the NCBI GenBank datasets and included 71 genomes from *V. crassostreae* and *V. gigantis* strains, as well as refseq genomes of 120 other species in the genus *Vibrio*. The fragLen parameter was set to 1200 (default 3000), as longer fragment sizes negatively affect ANI accuracy [28]. Prokka-annotated protein sequences were used in a preliminary standalone blast-2.4.0+ search against the core dataset (downloaded 21 October 2022) of virulence factor database VFDB [29] to identify putative genes for VFs in the draft genome of the *V. gigantis* isolate C24. This Whole Genome Shotgun project has been deposited in GenBank under Accession No. JAQGFV000000000.

### 2.8. Antibiotic Susceptibility Assay

The Kirby–Bauer’s disc diffusion method was used to determine the antibiotic susceptibility of *V. gigantis* C24 [30]; the method was used as described in the Clinical and Laboratory Standards Institute [31,32]. A total of fourteen antibiotics were selected, which were commercially used in the treatment of bacterial diseases [33,34,35] and recommended for use in disc diffusion tests by the CLSI [36] for *Vibrio* spp. The test isolate was grown on TSA media supplemented with 1.5% NaCl at 22 °C for 24 h. Then, a single colony of *V. gigantis* C24 was transferred to Mueller–Hinton broth (Merck-Millipore, Darmstadt, Germany), and the concentration was then adjusted to 0.5 MacFarland. The inoculum was streaked over the surface of Mueller–Hinton agar (Merck-Millipore, Darmstadt, Germany) plates with a sterile cotton swab. The selected antibiotics were ampicillin (10 µg), amoxicillin/clavulanic acid (30 µg), tetracycline (30 µg), chloramphenicol (30 µg), streptomycin (10 µg), gentamicin (10 µg), kanamycin (30 µg), nalidixic acid (30 µg), ciprofloxacin (5 µg), imipenem (10 µg), sulphamethox/trimethoprim (25 µg), cefotaxime (30 µg), tilmicosin (15 µg), and levofloxacin (5 µg). These discs were obtained from Oxoid (GmbH, Wesel, Germany) and Bioanalyse^®^ ASD (Bioanalyse Limited, Yenimahalle-Ankara/Türkiye), respectively. Standard antibiotic discs were placed in the medium at regular intervals using sterile forceps and incubated at 22 °C for 24 h. The zone diameters formed after incubation were measured. The results were evaluated based on CLSI breakpoints, specific for *Vibrio* spp. [36]: document M45-A2 for ampicillin, amoxicillin/clavulanic acid, tetracycline, chloramphenicol, gentamicin, Trimethoprim/Sulfamethoxazole, levofloxacin, cefotaxime, ciprofloxacin, and imipenem. However, the following CLSI breakpoints of *Enterobacteriaceae* were used when CLSI breakpoints were not available for *Vibrio* spp. [37]: document M100-S27 for streptomycin, kanamycin, and nalidixic acid. Other interpretive criteria [38] were used for tilmicosin, as no CLSI breakpoints were available. *E. coli* ATCC 25922 was used as a reference and quality control organism for antibacterial susceptibility testing [31,32,39].

### 2.9. Pathogenicity Study

#### 2.9.1. Preparation of the Bacterial Suspension

The recovered *V. gigantis* isolate C24 was cultured overnight in TSB (Tryptic Soy Broth) supplied with 1.5% NaCl at 22 °C. Bacterial cultures were harvested via centrifugation and resuspended in a sterile saline solution. Next, bacterial pellets were washed twice with phosphate-buffered saline (PBS) before the optical density of bacterial suspensions was adjusted to 1 × 10^10^ CFU/mL. A ten-fold serial PBS dilution was performed, and the prepared inoculum concentration ranged from 1 × 10^9^ CFU/mL to 1 × 10^5^ CFU/mL.

#### 2.9.2. Experimental Challenge

A total of 550 seabass juveniles with an average initial weight of 74.06 ± 0.08 g (mean ± SD) and length of 18 ± 1.0 cm were acclimatized to the laboratory conditions for 14 days with continuous aeration. Before the experimental infection, ten fish were randomly selected for screening to ensure they were free from the challenged pathogen. Then, the remaining 540 fish were allocated into six experimental groups in a triplicate design (90 individuals per group). Five groups were intraperitoneally injected with 100 μL of the freshly prepared *V. gigantis* C24 isolate suspension at different concentrations using an insulin syringe (1 × 10^5^ CFU/mL, 1 × 10^6^ CFU/mL, 1 × 10^7^ CFU/mL, 1 × 10^8^ CFU/mL and 1 × 10^9^ CFU/mL). The last experimental group of fish was injected with 100 μL of sterile PBS and served as the control. After day 5, the fish were fed a commercially purchased ration daily. Fish in all groups were observed daily for 21 days to record mortalities, clinical signs, and gross lesions. Dead fish were removed daily from tanks, and mortalities were recorded throughout the 21-day observation period. Following the experimental challenge, bacteriological samples were obtained from fish livers to re-isolate the *V. gigantis* used for the challenge to confirm the cause of mortality. The mean lethal dose (LD50) value was calculated using the probit analysis (SPSS 17.0, Chicago, IL, USA). Water quality parameters (including pH: 8.2–8.4, temperature: 16.1–17.2 °C, dissolved oxygen: 7.4–7.6 mg L^−1^, salinity: 28.3–29.1 ppt, total ammonia: 0.011–0.013 mg L^−1^, and nitrite: 0.02–0.026 mg L^−1^) were recorded daily and maintained at optimal levels for seabass culture.

## 3. Results

### 3.1. Clinical Signs and Gross Pathology

**Figure 1:**
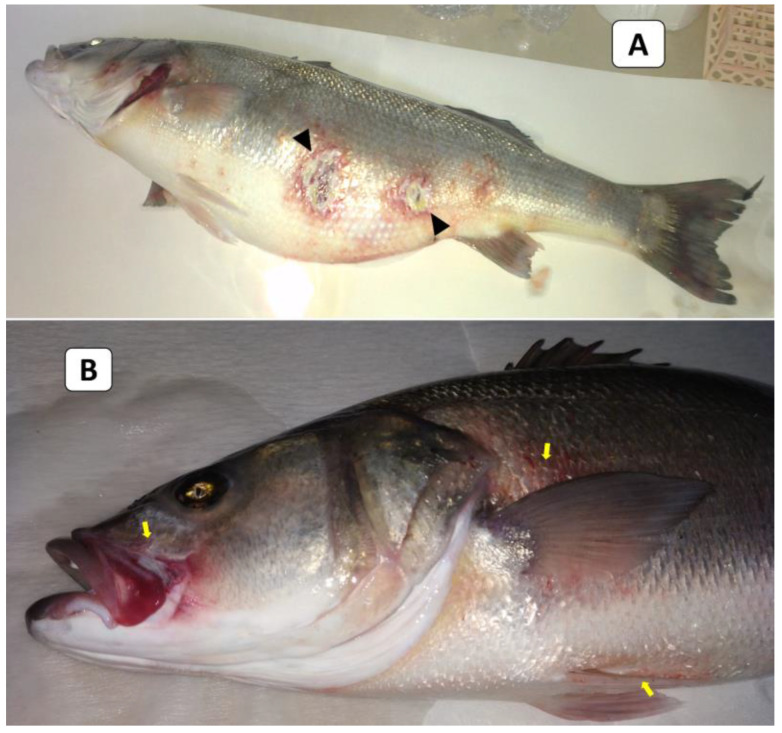
The clinical picture of naturally infected European seabass broodstock showed dermal ulcerations with hemorrhagic margins and distended abdomens (arrowheads; (**A**)) and hemorrhages below the pectorals, pelvic fins, and at the operculum (yellow arrows; (**B**)).

**Figure 2:**
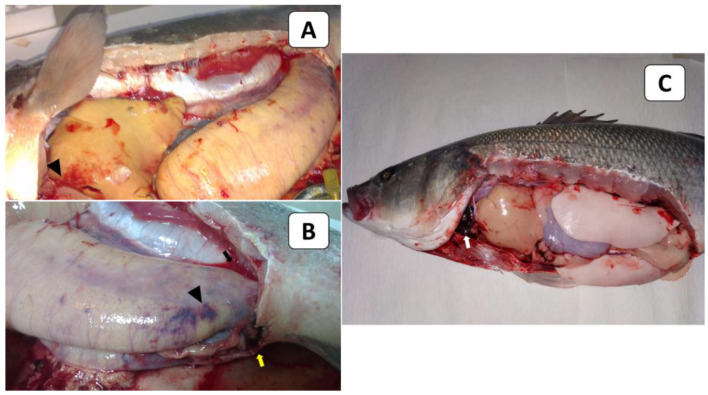
The PM findings of naturally infected European seabass broodstock showed liver congestion (arrowhead; (**A**)), congestion over the ovaries (arrowhead), hemorrhagic abdominal ascitic fluid (black arrow), congested hemorrhagic anal openings (yellow arrow; (**B**)), and congestion in the heart and gills (white arrow; (**C**)).

External signs of diseased European seabass included dermal ulcerations with hemorrhagic margins and distended abdomens (Figure 1A), as well as hemorrhages below the pectorals, pelvic fins, and at the operculum (Figure 1B). At the necropsy stage, the following effects were observed: liver congestion (Figure 2A), congestion over the ovaries, hemorrhagic abdominal ascitic fluid, congested hemorrhagic anal openings (Figure 2B), and congestion in the heart and gills (Figure 2C).

### 3.2. Histopathological Findings

**Figure 3:**
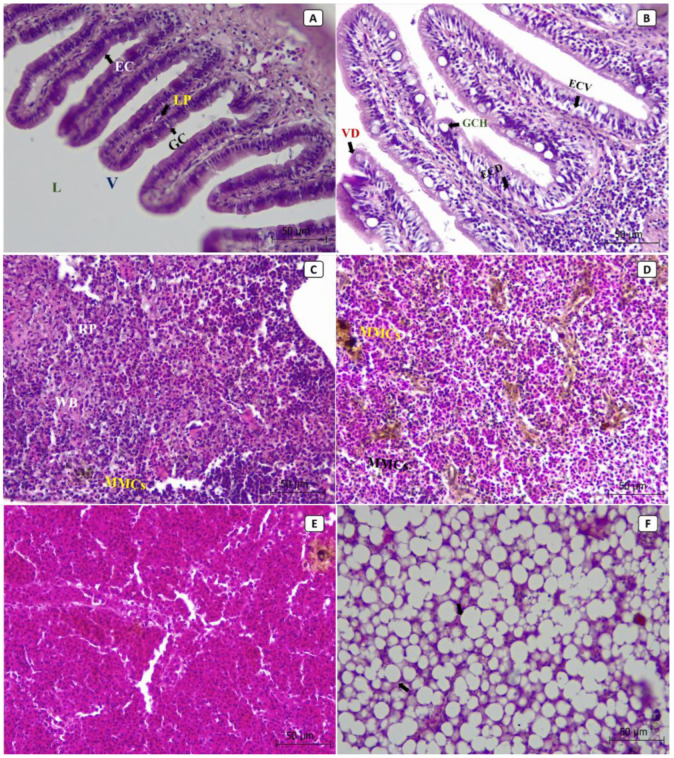
The photomicrographs in different tissues of European seabass broodstock (stained with H and E) showed the normal histological structure of the intestinal lumen (L), villus (V), epithelial cells (EC), goblet cells (GC), and lamina propria (LP) in normal, non-infected fish (**A**). In contrast, the intestinal tissues of the infected fish showed villus deformation (VD), goblet cell hypertrophy (GCH), epithelial cell deformation (ECD), and epithelial cell vacuolation (ECV) (**B**). The splenic tissues of the normal, non-infected fish showed normal structures of red pulp (RP) and white pulp (WP), as well as the aggregations of the melanomacrophage centers (MMCs) (**C**), while the splenic tissues of the infected fish showed an increased number and size of MMCs aggregations (**D**). The hepatopancreatic tissues of the normal, non-infected fish showed normal hepatocytes, nuclei, and hepatic sinusoids (**E**), while the hepatopancreatic tissues of the infected fish showed cytoplasmic vacuolization, severe fatty changes, pycnosis, local hemorrhages, and focal necrosis (**F**).

At the tissue level, most of the lesions were observed in the liver, spleen, and intestines of the diseased fish. The histopathological sections from non-infected control fish showed the normal histological structure of the intestinal lumen, villus, epithelial cells, goblet cells, and lamina propria (Figure 3A). At the same time, the splenic tissues showed the normal structure of red pulp, white pulp, and the aggregations of the melanomacrophage centers (MMCs) (Figure 3C). The hepatopancreatic tissues showed normal hepatocytes, nuclei, and hepatic sinusoids (Figure 3E). In contrast, the histopathological sections from diseased fish revealed that the most severely affected organ was the liver (Figure 3F), followed by the intestine (Figure 3B) and then the spleen (Figure 3D). In diseased fish, the intestinal sections revealed villus deformation, goblet cell hypertrophy, epithelial cell deformation, and vacuolation (Figure 3B). An increased number and size of MMCs aggregations were observed in splenic tissues (Figure 3D). Hepatopancreatic tissues showed cytoplasmic vacuolization, severe fatty changes, pycnosis, local hemorrhages, and focal necrosis (Figure 3F).

### 3.3. Bacteriological and Phenotypic Characterization

Three hundred and five motile, Gram-negative, curved rod-shaped bacterial isolates were obtained from the liver and spleen of diseased male and female fish. Bacterial growth was observed in the ovary samples of the female fish, while it was not observed in the gonad samples of the male fish. It was determined that all isolates had similar morphological and biochemical features (Table 1). Like the strains *V. gigantis* LPG13^T^ [16] and *V. gigantis* 915 [17], the isolates obtained in the present study were facultative anaerobes. They were positive for catalase, oxidase, indole, and arginine dihydrolase tests. Moreover, they produced NO_2_ and were sensitive to vibrio static agent O/129 (150 μg) (Table 1). In addition, 20 randomly selected isolates had the same 16S rRNA gene sequences. After confirming that the bacterial isolates belonged to the same species, we continued other identification analyses with the strain defined as C24. On TCBS agar plates, the retrieved bacterial isolate C24 formed green convex colonies 3–4 mm in diameter after 48 h of incubation at 22 °C (Figure S1). Under the same culture conditions, the bacterial colonies on TSA with 1.5% NaCl were 2–3 mm in diameter. Under a light microscope, the retrieved bacterial isolates were observed to be Gram-negative, motile, curved rod-shaped bacterium (Figure S1). Moreover, the API 20E profile is similar to that of the said strain, but our isolate can ferment sucrose (Table S1). The API 50CH test results revealed that the C24 strain can utilize GLU (D-glucose), GAL (D-galactose), MNE (D-mannose), FRU (D-fructose), MAN (D-mannitol), NAG (N-acetylglucosamine), ESC (esculin), CEL (D-cellobiose), MAL (D-maltose), TRE (D-trehalose), AMD (starch), and GLYG (glycogen) as sole carbon sources.

Three hundred and five motile, Gram-negative, curved rod-shaped bacterial isolates were obtained from the liver and spleen of diseased male and female fish. Bacterial growth was observed in the ovary samples of the female fish, while it was not observed in the gonad samples of the male fish. It was determined that all isolates had similar morphological and biochemical features (Table 1). Like the strains *V. gigantis* LPG13^T^ [16] and *V. gigantis* 915 [17], the isolates obtained in the present study were facultative anaerobes. They were positive for catalase, oxidase, indole, and arginine dihydrolase tests. Moreover, they produced NO_2_ and were sensitive to vibrio static agent O/129 (150 μg) (Table 1). In addition, 20 randomly selected isolates had the same 16S rRNA gene sequences. After confirming that the bacterial isolates belonged to the same species, we continued other identification analyses with the strain defined as C24. On TCBS agar plates, the retrieved bacterial isolate C24 formed green convex colonies 3–4 mm in diameter after 48 h of incubation at 22 °C (Figure S1). Under the same culture conditions, the bacterial colonies on TSA with 1.5% NaCl were 2–3 mm in diameter. Under a light microscope, the retrieved bacterial isolates were observed to be Gram-negative, motile, curved rod-shaped bacterium (Figure S1). Moreover, the API 20E profile is similar to that of the said strain, but our isolate can ferment sucrose (Table S1). The API 50CH test results revealed that the C24 strain can utilize GLU (D-glucose), GAL (D-galactose), MNE (D-mannose), FRU (D-fructose), MAN (D-mannitol), NAG (N-acetylglucosamine), ESC (esculin), CEL (D-cellobiose), MAL (D-maltose), TRE (D-trehalose), AMD (starch), and GLYG (glycogen) as sole carbon sources.

### 3.4. Bacterial Identification and Characterization

Full-length 16S rRNA and *gyrB* gene sequences obtained through NG-sequencing were analyzed to determine the taxonomical affiliation of bacterial isolate C24. The 16S rRNA gene sequence of this isolate was identical to that of *V. crassostreae* isolate 9CS106 (Accession No. CP016228.1) and strain ED295 (Accession No. CP064170.1). However, the present study’s isolate also had very high 16S rRNA gene similarity (99.9%) to three other species, namely *V. gigantis* strain LMG22741 (Accession No. AP025492.1), *V. coraliirubri* strain DSM27495 (Accession No. AP025470.1), and *V. artabrorum* strain CECT7226 (Accession No. AP025458.1). As the results were inconclusive, further online blast searches were performed with another housekeeping gene commonly used for vibrio species identification. The C24 isolate had 99.3% and 98.8% *gyrB* gene similarity to *V. gigantis* strain ACE001 (Accession No CP082384.1) and strain LMG 22741 (Accession No. AP025492.1), respectively. In comparison, the highest similarity to a *V. crassostreae gyrB* sequence was 97.8% (strain LMG 22240, Accession No. AP025476.1), which suggested that C24 is a *V. gigantis* isolate.

For reliable species identification, we had to perform average nucleotide identity (ANI) calculations using draft genome sequences. High whole-genome average nucleotide identity values (ANI > 97.7%) against four *V. gigantis* strain genomes unambiguously identified the *V. gigantis* C24 isolate as a member of this species. Moreover, ANI values calculated against 68 *V. crassostreae* strains and refseq genomes of 120 other vibrio species were notably lower (Table 2). A preliminary search for virulence factors in the draft genome of the *V. gigantis* isolate C24 identified several putative genes. For example, the genome of the *V. gigantis* isolate C24 encodes at least three exotoxins, including two aerolysins (genes aerA_1 and aerA_2) and a thermolabile hemolysin (Table 3).

### 3.5. Susceptibility to Antibiotics

The results of antibiotic susceptibility testing of *V. gigantis* isolate C24 against 14 kinds of antibacterial agents are described in Table 4. The disc diffusion assay values for the reference strain of *E. coli*, ATCC 25922, were within the quality control range specified by CLSI [31,32] and USA [39]. The *V. gigantis* isolate C24 was sensitive to amoxicillin/clavulanic acid (18 mm zone diameter), tetracycline (25 mm zone diameter), chloramphenicol (27 mm zone diameter), gentamicin (18 mm zone diameter), nalidixic acid (22 mm zone diameter), ciprofloxacin (30 mm zone diameter), imipenem (25 mm zone diameter), Trimethoprim/Sulfamethoxazole (28 mm zone diameter), cefotaxime (28 mm zone diameter), and levofloxacin (33 mm zone diameter). This bacterial strain was resistant to ampicillin (10 mm zone diameter), kanamycin (13 mm zone diameter), streptomycin (0 mm zone diameter), and tilmicosin (10 mm zone diameter).

### 3.6. Challenge Trial Results

**Figure 4:**
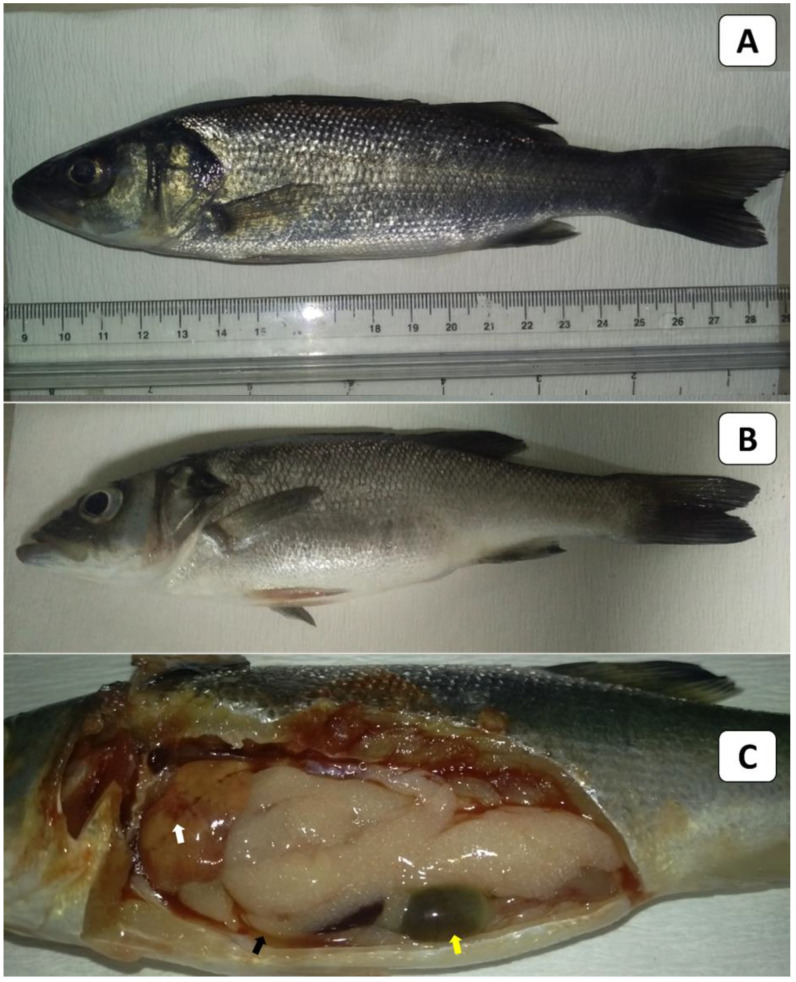
Clinical picture (external signs and PM lesions) in European seabass used in the LD50 testing. (**A**) shows the non-infected control fish, while (**B**,**C**) show the experimentally challenged fish. The fish in (**B**) showed slightly distended abdomens, and those in (**C**) showed mottled congested liver (white arrow), serous ascitic fluid (black arrow), and engorged gall bladders (yellow arrow).

European seabass in the control group (Figure 4A) showed no clinical signs or external lesions. In contrast, the experimentally challenged fish exhibited lethargy and a loss of appetite. The external signs of infected European seabass were in the form of slightly distended abdomens (Figure 4B). The PM lesions of the infected fish included abdominal ascitic fluid (arrow), mottled congested liver (arrow), and engorged gall bladders (arrow) (Figure 4C). It should be noted that some challenged fish died without any obvious clinical signs or external/internal lesions. Regarding the fish mortalities post-challenge, it was noticed that they started within 24 h post-infection in all experimentally infected fish groups. Moreover, during the 21-day observation period, fish mortalities occurred in a clear dose-dependent manner (Table 5). Notably, no mortalities were recorded in the control group, where fish had been injected with sterile PBS instead of live bacteria. An examination of the re-isolated bacteria revealed identical morphological and biochemical traits and 16S rDNA analysis results to the *V. gigantis* C24 isolate used in the challenge trial. The calculated LD50 of the bacterial isolate was 5.6 × 10^7^ CFU /mL (Figure S2).

## 4. Discussion

The present study reports a natural infection with *V. gigantis* that was first described in hatchery-reared European seabass broodstock and associated with mortalities during cold temperatures in the winter season. Indeed, *V. gigantis* and other members of the Splendidus clade, such as *V. cyclitrophicus*, have often been identified more in colder months [21]. In the present study, we hypothesized that the cold weather may act as a potential stress factor that triggers the infection with the *V. gigantis* strain and increases its virulence. However, this hypothesis has not been confirmed and requires further studies. Moreover, *V. gigantis* has been previously reported to peak during early spring in olive flounder-farmed sites on Jeju Island [19]. In addition, a recently published study demonstrated that *V. gigantis* was identified frequently in the spring season in gilthead seabream in Greece [21]. This may support our claim that *V. gigantis* may become a problem for the aquaculture industry not only in Türkiye but also in other South European countries in colder seasons of the year. Nonetheless, further surveys and epidemiological studies are still required to confirm the existence of pathogenic strains in several countries.

The causative agent, *V. gigantis*, was originally isolated from the hemolymph of cultured Pacific oyster [16] and later from *A. japonicus* [17] and *H. arguinensis* [18]. It has also been reported in three finfish species, namely the olive flounder *P. olivaceus* [19], the dark-banded rockfish *S. inermis* [20], and gilthead seabream [21]. The recorded clinical signs and PM lesions in the present study in the infected European seabass were in the form of hemorrhagic septicemia. These findings were similar to those previously noted in dark-banded rockfish [20]. Unlike the present study, these authors observed mixed infections with *V. gigantis* and concluded that the known fish pathogen *V. harveyi* was solely responsible for the clinical signs (skin ulcers and tail rot) and PM lesions (liver discoloration, yellowish fluid in intestines and ascites) they observed. In the present study, several histopathological changes with varying degrees were observed in the livers, intestines, and spleen of the naturally infected European seabass broodstock. To our knowledge, no previously published studies have reported histopathological changes in finfish infected with *V. gigantis*. However, these findings may be associated with the virulence factors present in this bacterial strain.

The colony appearance on TCBS agar and the morphological characteristics of *V. gigantis* isolate C24 were similar to those reported for the *V. gigantis* isolate from dark-banded rockfish [20]. They were also similar to *V. gigantis* isolates from invertebrates of the Sea of Japan [17]. Both *V. gigantis* and *V. crassostreae* were originally isolated from *C. gigas* [16,40]. They are closely related species and cannot be separated by biochemical testing alone, as strains display some variation in their metabolic abilities. For example, some differences exist in the phenotypic traits of our *V. gigantis* C24 isolate and *V. gigantis* strains isolated from other aquatic organisms. Differences in the ability of *V. gigantis* strains to grow on culture media supplemented with varying salt concentrations have also been reported previously [41]. This may be attributed to the genetic variation among bacterial strains, temperature requirements for bacterial growth, the fish host species from which the bacterium has been isolated, geographical localities, or other factors [16,17,41]. Thus, these variable features may lead to the misidentification of *Vibrio* isolates [42]. Interestingly, the results of carbohydrate assimilation of C24 strain of the present study were similar to those reported in the *V. gigantis* strain isolated from *C. gigas* [16], confirming the biochemical characteristics of our isolate.

Several species in the genus *Vibrio* cannot easily be distinguished between using full-length 16S rRNA gene sequences or single house-keeping genes like the *gyrB* gene. For example, a phylogenetic tree based on the 16S rRNA gene sequences did not allow for a clear differentiation between two representative isolates of *V. gigantis* (LGP 13T and LGP 37) and other species phenotypically related to *V. splendidus* [16]. However, the researchers of this study reported that a phylogenetic tree based on *gyrB* gene sequences could help to distinguish them from their closest phylogenetic neighbors [16]. Seventeen years later, with more available data, the results from sequence analysis with these two commonly used genes for species identification were inconclusive. Therefore, the taxonomic affiliation of the retrieved bacterial isolate C24 had to be verified via whole-genome ANI calculations, as recommended previously [43]. The calculated ANI value between the bacterial C24 isolate and the *V. gigantis* type strain is well above the commonly accepted species (ANI >95%) demarcation limit [44,45], thus, the isolate was unambiguously as *V. gigantis*.

Antibiotics and antimicrobials are extensively used to prevent or control bacterial diseases that may challenge the aquaculture species. In the present study, the bacterial isolate C24 was sensitive to several antimicrobials. However, it was resistant to ampicillin, kanamycin, streptomycin, and tilmicosin. These data are important for constructing a strategy for controlling this bacterial infection and avoiding the development of antibiotic resistance [46]. The development of bacterial resistance may be associated with the presence of resistant genes [47]. Thus, further research studies should be carried out to elucidate the genes encoding for the resistance of this bacterial isolate to antibiotics.

The pathogenic potential of *V. gigantis* is unclear, and this vibrio species has recently been referred to as non-pathogenic or low virulent [19]. However, the gross pathology and histopathological alterations in naturally infected European seabass broodstock and experimentally challenged fish provide compelling evidence that the *V. gigantis* C24 isolate has pathogenic potential. It is, however, worth noting that the clinico-pathological picture revealed decreased virulence in the experimentally infected seabass compared to naturally infected broodstock. This means that the C24 isolate may be less virulent in juvenile seabass. Notably, the fish developmental stage (size/age) and immune status may be the main reasons behind these discrepancies. Conditions are after all different in indoor experimental tanks compared to an open-air culture system. A different infection route/dose would likely lead to a faster progression of the experimentally induced disease, which also could affect the clinico-pathological picture.

It is well known that identifying virulence factors is important for evaluating bacterial pathogenicity because these factors will allow bacteria to infect and damage the host fish [9]. Herein, the present study showed another line of evidence that the draft genome of isolate C24 encodes three exotoxins (two aerolysins and a thermolabile hemolysin). For example, an aerolysin-like cytolytic toxin from *V. splendidus* was suspected of being involved in intestinal tract damage and mortalities in turbot and cod larvae [48]. Moreover, a thermolabile hemolysin (TLH) from *V. alginolyticus* has been shown to induce membrane vesiculation, apoptosis, and post-apoptotic necrosis in sea bream erythrocytes [49]. These putative virulence genes in the C24 genome likely encode exotoxins with similar functions. The pathogenic potential of the C24 may be linked to the presence of these virulence genes. Continued research, including comparative genomics, is underway to better understand the pathogenic potential of the C24 isolate and other *V. gigantis* strains.

## 5. Conclusions

The present study reported the first case of *V. gigantis* infection in European seabass (hatchery-reared broodstock), leading to notable mortality during the cold winter season. The observations made during our research and experiment involving the *V. gigantis* isolate C24 provide the first pieces of solid evidence that this bacterium has pathogenic potential in an economically important farmed finfish species. Although the whole-genome ANI values confirmed the bacterial strain identity, a full genomic analysis of the sequenced *V. gigantis* C24 isolate is required to better understand its pathogenic potential. To put it briefly, our findings suggest that *V. gigantis* is an emerging pathogen in Turkish and possibly Mediterranean aquaculture. Taken together, the findings and results of this study should merit international interest, as this vibrio species could become responsible for economic losses in European aquaculture. To address this issue, the importance of a proper surveillance protocol, epidemiological data collection, and appropriate control measures should be emphasized.

## Figures and Tables

**Table 1:** Phenotypic characterization of *Vibrio gigantis* isolates.

Characteristics	Vibrio Isolate—This Study	*V. gigantis* 915 [17]	*V. gigantis* [16]
Morphology	curved rod	rod	curved
Motility	+	+	+
Gram staining	Negative	Negative	Negative
Oxidase	+	+	+
Catalase	+	+	+
Indole	+	+	+
Oxidation/fermentation	+/+	+/+	+/+
Arginine dihydrolase	+	+	+
Lysine decarboxylase	−	−	−
Ornithine decarboxylase	−	−	−
Simmons citrate	−	−	−
Nitrate reduction	+	+	+
Sensitivity to: 0129 (10/150 µg)	+	+	+
Growth on TCBS agar Colony color	+ Green	+ Green	+ Yellow
Growth at:			
4 °C	+	+	+
30 °C	+	−	ND
35 °C	−	−	−
Growth at:			
0% NaCI	−	−	−
2% NaCI	+	ND	+
4% NaCI	+	ND	+
6% NaCI	+	+	+
8% NaCI	+	+	−
10% NaCI	−	−	ND

(−) = negative; (+) = positive; (ND) = Not detected.

**Table 2:** Whole-genome average nucleotide identity (ANI) values for vibrio isolate C24 from European seabass broodstock against the eight highest scoring strains in a comprehensive dataset comprising refseq genomes of 122 known species in genus *Vibrio*.

Query Strain ID	Refseq Strain ID	NCBI Accession No.	ANI Value (%)
Vibrio isolate C24 (this study)	*Vibrio gigantis* LGP 13T	NZ_MVJE01000001.1	98.0
	*Vibrio gigantis* strain CCUG56969T	NZ_VXJS01000001.1	97.9
	*Vibrio gigantis* strain ACE001	NZ_CP092384.1	97.9
	*Vibrio gigantis* strain 43_P_281	NZ_JAGDQE010000001.1	97.7
	*Vibrio crassostreae* strain 16SF1_51	NZ_RJKJ01000001.1	90.9
	*Vibrio celticus* strain Rd 8.15	NZ_MVJF01000001.1	90.8
	*Vibrio coralliirubri* strain Evd3	NZ_ORXW01000001.1	90.6
	*Vibrio bathopelagicus* strain Sal10	NZ_CP062500.1	90.3

FastANI software calculations unambiguously identified the C24 isolate as *V. gigantis*.

**Table 3:** Results from a preliminary search for virulence factor genes in the draft genome of the *V. gigantis* isolate C24 from European seabass broodstock.

Prokka Locustag/Annotation	Pident (%)	e-Value	Bitscore	VF Database Descript./ID/Source Bact.
C24_00250 Thermolabile hemolysin	63.0	0	571	(tlh) thermolabile hemolysin TLH (VF0610) *Vibrio cholerae* O1 biovar El Tor str. N16961
C24_04078 Aerolysin gene (aerA_2)	53.4	0	533	(aerA/act) Aerolysin A (VF0481) *Aeromonas hydrophila* ML09-119
C24_02991 Aerolysin gene (aerA_1)	43.2	1.03E-148	431	(aerA/act) Aerolysin A (VF0481) *Aeromonas hydrophila* ML09-119

The protein sequence identity (pident) and blastp search e-values and bitscores are included. Large bitscores show that these hits are significant and indicate homology between proteins.

**Table 4:** Susceptibility of the *V. gigantis* isolate C24 against 14 kinds of antibacterial agents.

Antibiotic Discs	Abbreviation	Content (μg)	Diameters of Inhibition Zone (mm)	Susceptibility
Ampicillin	(AMP)	10	10	R
Amoxicillin/clavulanic acid	(AMC)	30	18	S
Tetracycline	(TET)	30	25	S
Chloramphenicol	(CHL)	30	27	S
Streptomycin	(ST)	10	0	R
Gentamicin	(CN)	10	18	S
Kanamycin	(K)	30	13	R
Nalidixic acid	(NA)	30	22	S
Ciprofloxacin	(CIP)	5	30	S
Imipenem	(IPM)	10	25	S
Trimethoprim/Sulfamethoxazole	(SXT)	25	28	S
Cefotaxime	(CTX)	30	28	S
Tilmicosin	(TIL)	15	10	R
Levofloxacin	(LEVO)	5	33	S

Abbreviations: R, Resistant; S, Sensitive.

**Table 5:** Daily and cumulative mortalities recorded in European seabass injected intraperitoneally with different concentrations of *V. gigantis* during a 21-day observation period.

Groups		Days Post-Challenge					Total No. of Challenged Fish	Total No. of Dead Fish	Mortality (%)
		1st	2nd	3rd	4th	5th–20th			
Group 1	Control (PBS)	0	0	0	0	0	90	0	0
Group 2	1 × 10^5^ CFU/mL	2	2	0	0	0	90	4	4.4
Group 3	1 × 10^6^ CFU/mL	6	5	3	2	0	90	16	17.8
Group 4	1 × 10^7^ CFU/mL	15	9	6	1	0	90	31	34.4
Group 5	1 × 10^8^ CFU/mL	32	10	2	2	2	90	48	53.3
Group 6	1 × 10^9^ CFU/mL	55	19	13	2	1	90	90	100

## Data Availability

The datasets generated during the present study are available from the corresponding author upon reasonable request.

## Supplementary Materials

The following supporting information can be downloaded at: https://www.mdpi.com/article/10.3390/ani13223580/s1, Figure S1. (A) Green convex shiny colonies of 3–4 mm in diameter were grown on the TCBS agar plates. These colonies were obtained from pure culture of the suspected bacterium that isolated from naturally infected European seabass (*Dicentrarchus labrax*) broodstock. (B) Light microscopic capture of a gram-negative curved rod-shaped bacterium isolated from naturally infected European seabass broodstock; Figure S2. Probit graphic method for LD50 estimation; Table S1. The API 20E test results of the bacterial isolate *Vibrio gigantis* C24 of the present study, *V. crassostreae* isolates (19B and LGP 7T), and *V. gigantis* LGP 13T type strain.
