# Supplementary material for: Isolation, Identification, and Pathogenicity of Vibrio gigantis Retrieved from European Seabass (Dicentrarchus labrax) Farmed in Türkiye
Source: Animals (Basel). 2023 Nov 20;13(22):3580. doi: 10.3390/ani13223580 (PMC10668765; doi:10.3390/ani13223580)
Supplement: Supplementary file 1 [file animals-13-03580-s001.zip › animals-2683635-supplementary.pdf]

Table S1. The API 20E test results of the bacterial isolate *Vibrio gigantis* C24 of the present study, *V. crassostreae* isolates (19B and LGP 7T), and *V. gigantis* LGP 13T type strain.

API 20 E results	19B	LGP 7T	C24	LGP 13T
**ONPG**	+	-	-	-
**ADH**	+	-	+	+
**LDC**	-	-	-	-
**ODC**	-	-	-	-
**CIT**	-	-	-	n.a.
**H_2S**	-	-	-	n.a.
**URE**	-	-	-	-
**TDA**	-	-	-	n.a.
**IND**	+	+	+	+
**VP**	-	-	-	n.a.
**GEL**	+	+	+	+
**GLU**	-	+	+	+
**MAN**	-	+	+	+
**INO**	-	-	-	-
**SOR**	-	-	-	-
**RHA**	-	-	-	-
**SAC**	-	+	+	-
**MEL**	+	+	+	+
**AMY**	+	+	+	+
**ARA**	-	-	-	-
**Presumptive ID**				
**API results**	*V. fluviais*	*V. fluviais*	*V. fluviais*	n.a.
**Genetic identification**	*V. crassostreae*	*V. crassostreae*	*V. gigantis*	*V. gigantis*
**References**	(Tomasoni et al., 2022)	(Faury et al., 2004)	The present study	(Le Roux et al., 2005)

Differences between the C24 isolate and *V. gigantis* type strain is highlighted in grey; The abbreviations: ONPG: β-galactosidase enzyme test, ADH: arginine dihydrolase, LDC: lysine decarboxylase, ODC: ornithine decarboxylase, CIT: citrate, H2S: hydrogen sulfide, URE: urease, TDA tryptophan deaminase, IND: Indole test, VP: the Voges-Proskauer test, GEL: gelatinase test, fermentation of GLU: glucose, MAN: mannose, INO: inositol, SOR: sorbitol, RHA: rhamnose, SAC: sucrose, MEL: melibiose, AMY: amygdalin and ARA: arabinose, n.a.: data not available.

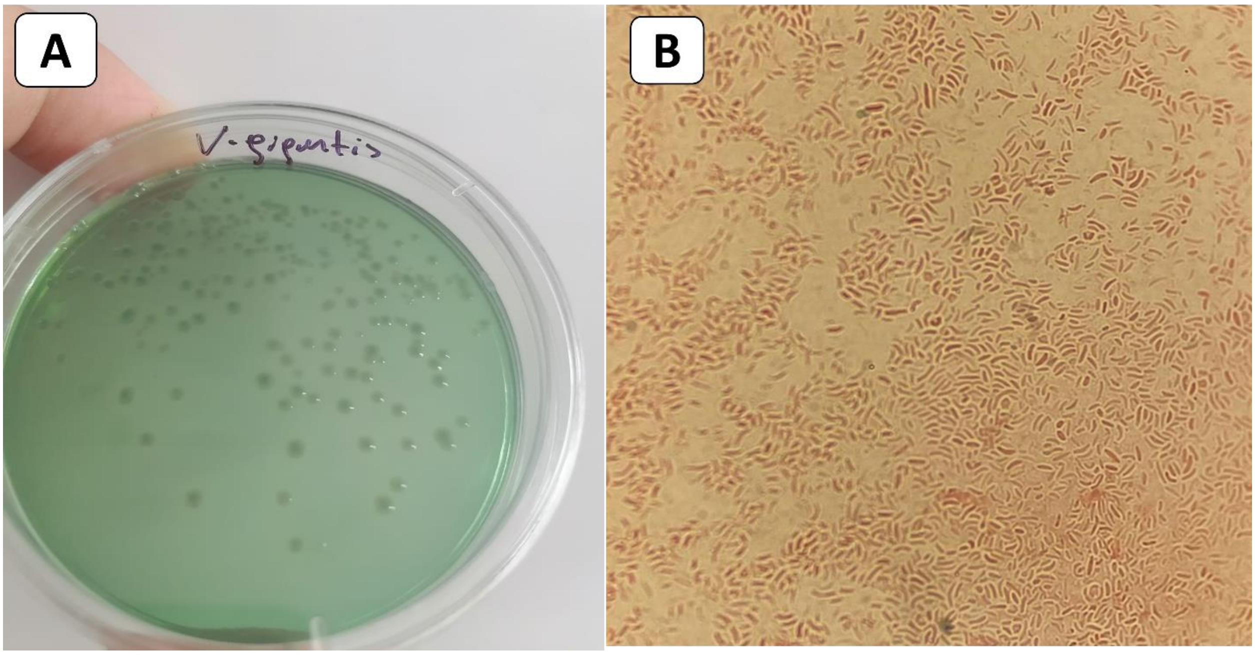

Figure S1. (A) Green convex shiny colonies of 3–4 mm in diameter were grown on the TCBS agar plates. These colonies were obtained from pure culture of the suspected bacterium that isolated from naturally infected European seabass (*Dicentrarchus labrax*) broodstock. (B) Light microscopic capture of a gram-negative curved rod-shaped bacterium isolated from naturally infected European seabass broodstock.

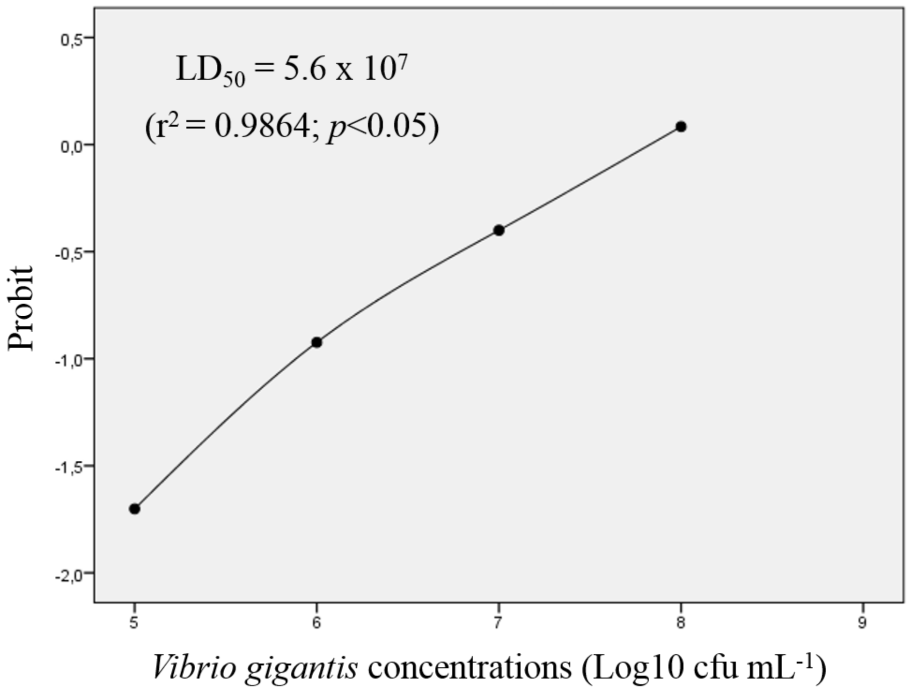

Figure S2. Probit graphic method for LD50 estimation.
